# Roles of pH and phosphate in rare earth element biosorption with living acidophilic microalgae

**DOI:** 10.1007/s00253-024-13068-8

**Published:** 2024-03-14

**Authors:** Jens Kastenhofer, Oliver Spadiut, Vladimiros G. Papangelakis, D. Grant Allen

**Affiliations:** 1 Department of Chemical Engineering and Applied Chemistry, University of Toronto, 200 College Street, Toronto, ON M5S 3E5 Canada; 2 Institute of Chemical, Environmental and Bioscience Engineering, Research Division Biochemical Engineering, Research Group Integrated Bioprocess Development, TU Vienna, Gumpendorfer Straße 1a, 1060 Vienna, Austria

**Keywords:** Rare earth elements, Biosorption, Bioaccumulation, Acidophiles, *Galdieria sulphuraria*, Algal biomass

## Abstract

**Abstract:**

The increasing demand for rare earth elements (REEs) has spurred interest in the development of recovery methods from aqueous waste streams. Acidophilic microalgae have gained attention for REE biosorption as they can withstand high concentrations of transition metals and do not require added organic carbon to grow, potentially allowing simultaneous sorption and self-replication of the sorbent. Here, we assessed the potential of *Galdieria sulphuraria* for REE biosorption under acidic, nutrient-replete conditions from solutions containing ≤ 15 ppm REEs. Sorption at pH 1.5–2.5 (the growth optimum of *G. sulphuraria*) was poor but improved up to 24-fold at pH 5.0 in phosphate-free conditions. Metabolic activity had a negative impact on REE sorption, additionally challenging the feasibility of REE biosorption under ideal growth conditions for acidophiles. We further examined the possibility of REE biosorption in the presence of phosphate for biomass growth at elevated pH (pH ≥ 2.5) by assessing aqueous La concentrations in various culture media. Three days after adding La into the media, dissolved La concentrations were up to three orders of magnitude higher than solubility predictions due to supersaturation, though LaPO_4_ precipitation occurred under all conditions when seed was added. We concluded that biosorption should occur separately from biomass growth to avoid REE phosphate precipitation. Furthermore, we demonstrated the importance of proper control experiments in biosorption studies to assess potential interactions between REEs and matrix ions such as phosphates.

**Key points:**

*• REE biosorption with G. sulphuraria increases significantly when raising pH to 5*

*• Phosphate for biosorbent growth has to be supplied separately from biosorption*

*• Biosorption studies have to assess potential matrix effects on REE behavior*

**Supplementary Information:**

The online version contains supplementary material available at 10.1007/s00253-024-13068-8.

## Introduction

Rare earth elements (REEs) are essential components of many advanced technology products, such as consumer electronics, clean energy technologies, catalysts, and defense systems. As demand for these critical minerals is increasing, it is crucial to develop new methods to recover REEs from secondary sources, such as wastes from mining, metallurgical processes, or end-of-life electronics (Dev et al. 2020; Pawar and Ewing 2022). Conventional extraction methods based on organic solvents have limited applicability to such waste streams, as the REE concentrations are usually too low for solvent extraction to be efficient (< 100 ppm) (Opare et al. 2021).

Microorganisms have gained considerable attention for the extraction of REEs from dilute aqueous solutions, as they have been shown to grow and perform well in low metal concentration environments and microbial biomass can be produced at low cost in an environmentally benign way (Dev et al. 2020; Mattocks and Cotruvo 2020; Pinto et al. 2023). The extraction process happens largely by adsorption of the metal cations on the cell surface and is commonly referred to as biosorption. It is driven by the high concentration of negatively charged binding sites (carboxyl, hydroxyl, amine, and phosphate groups) in the cell wall. Ion exchange, chelation, or precipitation are commonly reported binding mechanisms. One of the major chemical factors influencing biosorption is the solution pH, since the functional groups of the cell wall are easily protonated with decreasing pH, whereas high pH leads to insoluble REE hydroxide formation. Biosorption is therefore commonly preferred at mildly acidic conditions (pH 3–6), with a common optimum reported at pH 5 (Mattocks and Cotruvo 2020).

While biosorption does not require the cells to be metabolically active or alive (in contrast to cellular bioaccumulation (Diep et al. 2018)), the use of living microorganisms for REE recovery offers a significant advantage in that it could allow self-replication of the sorbent in sequence with or even during the sorption process. Acidophilic microalgae are deemed particularly suited for this due to their high metal tolerance in the acidic nature of most aqueous metal-bearing waste streams (e.g., leachates or acid mine drainage), and the use of CO_2_ instead of organic carbon as a substrate for biomass generation (Minoda et al. 2015; Kim et al. 2022; Sun et al. 2022; Zak et al. 2023). One notable example is *Galdieria sulphuraria*, which is reported to have removed over 90% of REEs from a dilute (5 ppm) metal solution under highly acidic conditions (pH 1.5–2.5) (Minoda et al. 2015).

In order to harness the full potential of a living, self-replicating biosorbent, nutrients for its proliferation must be provided. Phosphorus is one of the essential micronutrients and is usually encountered and supplied as orthophosphate in biological processes. However, lanthanide phosphates have an extremely low solubility constant (− log *K*_sp_ ≈ 25–26) (Firsching and Brune 1991) and precipitation of REE phosphates is often reported to occur at pH higher than 2 or 3 (Beltrami et al. 2015; Zhi et al. 2020). This presents a dilemma for REE biosorption with living microorganisms. On one hand, extremely acidic conditions ensure REE solubility but impede adsorption. On the other hand, mildly acidic to neutral conditions are favorable for adsorption, but lead to the removal of phosphates, which are required for the growth of the biosorbent, from the solution, due to REE phosphate precipitation.

In the present study, we explored the potential of *G. sulphuraria* to biosorb REEs under acidic, nutrient-replete conditions that allow simultaneous replication of the biosorbent. To this end, we first aimed to reproduce the results reported by Minoda et al. (2015) and subsequently conducted sorption experiments in growth media spiked with REEs. We then explored the feasibility of REE biosorption in systems containing phosphate at various acidity levels through thermodynamic simulations coupled with experimental verification.

## Materials and methods

### Algal cultures

Axenic cultures of *Galdieria sulphuraria* SAG 21.92 (SAG) were grown in a liquid mineral medium described by Abiusi et al. (2021), with the modification that we omitted EDTA and iron was supplied as Fe_2_SO_4_ (herein referred to as *Galdieria* medium). Initial pH (denoted as pH_0_) was adjusted with H_2_SO_4_ to 2.5, unless stated otherwise. For mixotrophic and heterotrophic cultures, the medium was supplemented with 25 mM glucose, unless stated otherwise. Cultures were grown in conical flasks in an orbital shaking incubator at 180 rpm. Temperature was set to 42 °C and CO_2_ was at atmospheric levels (~ 0.04%). Heterotrophic cultures were grown in the dark, while phototrophic and mixotrophic cultures were continuously illuminated by warm white LEDs. PAR light intensity was set to 100 µmol_ph_ m^−2^ s^−1^ and checked with an LI-180 handheld spectrometer (LI-COR).

### Biomass and optical density measurement

Biomass concentration was determined gravimetrically via filtration of cell suspension onto pre-weighed glass fiber filters (GA-55, Advantec) and subsequent drying and weighing. Optical density (OD) of cell suspensions was measured in 1-cm cuvettes at 750 nm with a DR3900 spectrophotometer (Hach).

### Biosorption experiments

With the goal to reproduce the results of Minoda et al. (2015), REE biosorption with living *G. sulphuraria* cells was first performed according to their study under mixotrophic or heterotrophic microoxic conditions, respectively, with slight modifications. The metal solutions in which biosorption was evaluated were composed of 5 ppm each of Cu (79 µM), La (36 µM), Nd (35 µM), and Dy (31 µM), supplied as sulfate salts, except for Dy, which was supplied as a chloride salt. pH was adjusted to 2.5 or 5.0, respectively, using H_2_SO_4_. Unlike in Minoda et al. (2015), no 2-(N-morpholino)ethanesulfonic acid (MES) buffer was used as a buffer at pH 5.0, since MES scavenges lanthanide ions by complexation (Mandal et al. 2022). Mixotrophic conditions were created by supplying 25 mM glucose and white light as described above. Microoxic conditions were tested based on Ahlf (1988), who showed that biosorption of transition metals with *G. sulphuraria* is affected by oxygen levels. To this end, heterotrophically grown *G. sulphuraria* cells were sparged with 99.998% N_2_ in media bottles sealed with rubber septa and cultivated them in the dark. Cells were adapted to microoxic conditions overnight in the presence of 25 mM glucose before sorption experiments. For biosorption under microoxic conditions, 25 mM acetic acid was supplied instead of glucose. To obtain biomass for biosorption, *G. sulphuraria* was grown either in mixotrophic or heterotrophic/microoxic mode as described above to OD 4, subsequently centrifuged at 5000 × g for 8 min at 21 °C, washed with ultrapure water, and resuspended in the metal mix to reach OD 10. Biosorption was performed in triplicate in 125-mL conical flasks (mixotrophic) or 100-mL capped media bottles (microoxic) at a reaction volume of 30 mL. Samples of 10 mL were taken after 3 h and 20 h, respectively, and centrifuged at 8000 × g for 5 min at 21 °C. Pellets were washed once with ultrapure water before biomass quantification. Supernatants were filtered through a 0.22-µm nylon filter and immediately diluted in 5% nitric acid before metal analysis via ICP-OES (Agilent 720 Series).

For REE biosorption with *G. sulphuraria* under nutrient-replete conditions, 100-mL algal cultures were prepared in conical flasks in *Galdieria* medium at pH 1.5 and 2.5, respectively. At pH 1.5, three different trophic modes were tested (phototrophic, mixotrophic, heterotrophic), while at pH 2.5, only phototrophic conditions were tested. Glucose for mixotrophic and heterotrophic conditions was supplied at 55 mM. A 10 mM La_2_(SO_4_)_3_ stock was spiked to duplicate cell suspensions to reach a concentration of 20 µM and 100 µM at pH 1.5 and 2.5, respectively, and the cultures were then incubated as described above according to their respective trophic mode. The biomass concentrations at which La was added and duration of the experiments (at least 6 days) are shown in the “Results” section. Samples taken at the beginning and end of each condition were processed and analyzed as in biosorption experiments described above.

Metal sorbed by the biomass *q*_Me_ was determined according to Eq. 1:

1 $${q}_{Me}=\frac{{c}_{Me,t}-{c}_{Me,0}}{{c}_{X,t}}$$

where *c*_Me,*t*_ and *c*_Me,0_ are the metal concentrations in the supernatant at time *t* and at the start of the experiment, respectively, and *c*_X,*t*_ is the biomass concentration at time *t.*

### REE phosphate solubility experiments

OLI StreamAnalyser v.11.5 (OLI Systems) with the Aqueous database was used to calculate precipitation points of La in *Galdieria* medium. A composition survey was performed at 25 °C with KH_2_PO_4_ ranging from 2 × 10^−5^ to 10^−2^ M and H_2_SO_4_ ranging from 10^−5^ to 2 × 10^−2^ M. In these simulations, the La salt was LaCl_3_ and the precipitate LaPO_4_ • x H_2_O. The thermodynamic data for LaPO_4_ • x H_2_O is from Liu and Byrne (1997).

La precipitation in *Galdieria* medium was also assessed experimentally. Medium was prepared as described above and pH was adjusted to 2.5 or 3.0 using H_2_SO_4_. One hundred times stock solutions of LaCl_3_ were spiked to 9.9 mL medium in 15-mL polypropylene tubes to final concentrations of 100 µM, 400 µM, 1000 µM, or 2000 µM. Approximately 1 mg of previously synthesized LaPO_4_ (see Supplementary Text 1) was added as crystallization seed. The solutions were incubated on a tube rotator for at least 72 h at room temperature before centrifugation at 10,000 × g for 5 min at 21°C. Supernatants were filtered through a 0.22 µm nylon filter and immediately diluted in 5% nitric acid before analysis via ICP-OES. Experiments were done in triplicate.

La solubility was tested in three additional culture media that have been previously used in biosorption studies: Bold’s basal medium (Fischer et al. 2019; Fritz et al. 2022a, b); Hutner medium (Kim et al. 2022); and a medium based on yeast extract (Kim et al. 2022). Bold’s basal medium was prepared according to Nichols and Bold (1965) with slight modifications (Table S1) and adjusted to pH 6.8 with NaOH. Hutner medium was prepared according to Kim et al. (2019), and the pH of the finished medium was measured to be 3.3. The yeast extract medium contained 5 g/L yeast extract, 55.5 mM glucose, and 0.25 mM KH_2_PO_4_, and pH was set to 3.3 with HCl. This phosphate concentration is based on the composition of the stream water used by Kim et al. (2022) for biosorption, assuming that all phosphorus is present as orthophosphate. In addition to these culture media, controls were prepared containing only KH_2_PO_4_ at the concentration present in the respective medium and HCl to set the pH to 3.3. A control containing only ultrapure water was also included. A 10 mM stock solution of LaCl_3_ was spiked to 9.9 mL medium or control solution in 15-mL polypropylene tubes to a final concentration of 100 µM. Reaction conditions, duration, and analysis were the same as in experiments with *Galdieria* medium described above.

## Results

### Biosorption of REEs under phosphate-free conditions

**Fig. 1:**
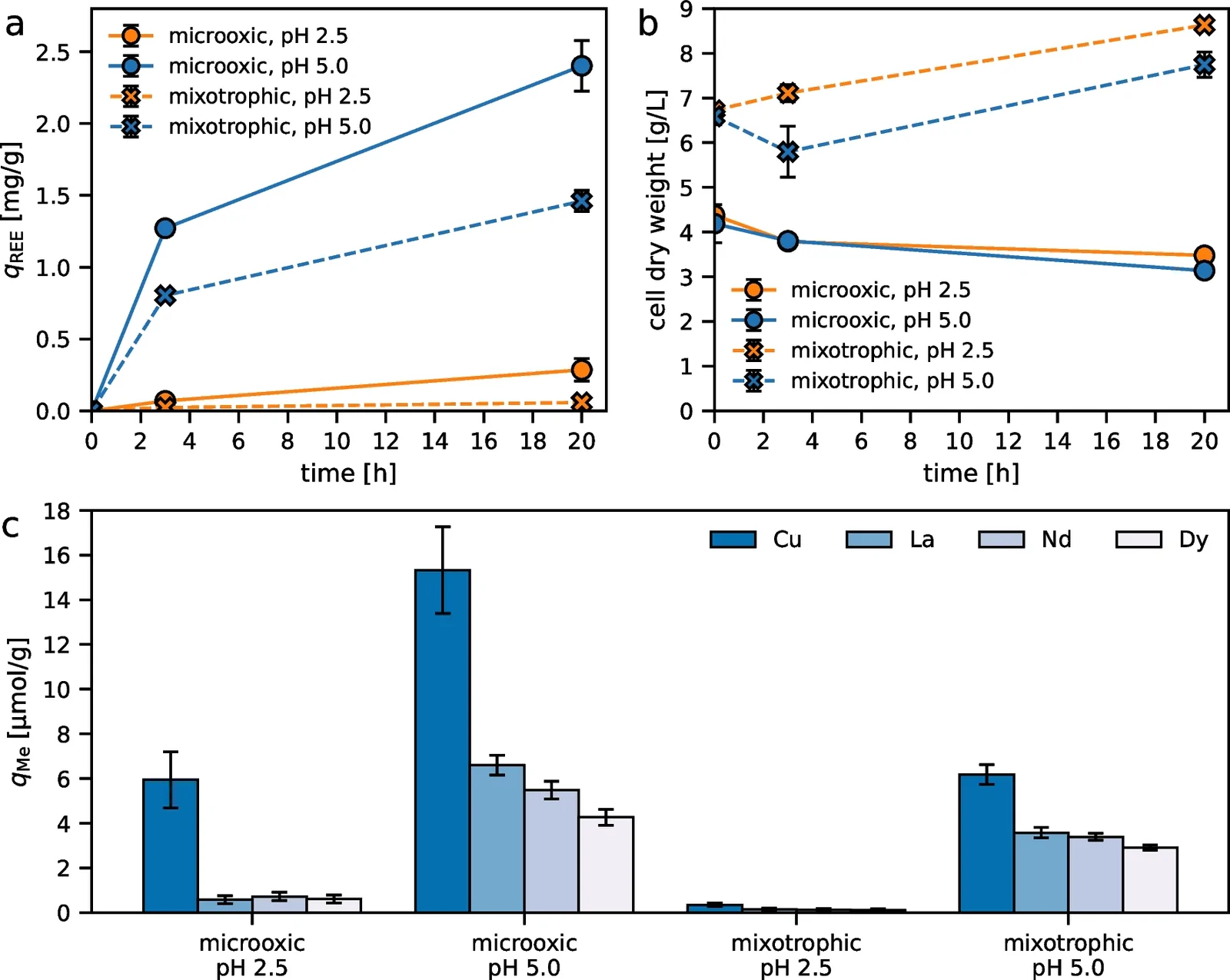
Metal biosorption with *G. sulphuraria* in an acidic sulfate system containing initially 5 ppm each of Cu (79 µM), La (36 µM), Nd (35 µM), and Dy (31 µM). Total sorbed REEs (**a**) and biomass concentration (**b**) at initial pH 2.5 or 5.0 and under microoxic or mixotrophic conditions. Individual metals sorbed after 20 h are shown in (**c**)

In order to confirm REE biosorption capabilities of *G. sulphuraria* under highly acidic conditions as reported by Minoda et al. (2015), we first performed experiments in an acidic sulfate system without nutrients except for carbon (mixotrophic or microoxic heterotrophic conditions). REE biosorption occurred over more than 3 h, increasing two to fourfold after 20 h (Fig. 1a). In total, biomass-specific REE sorption after 20 h at pH 2.5 was 0.06 mg/g and 0.29 mg/g under mixotrophic and microoxic conditions, respectively. Raising initial pH to 5.0 substantially increased REE sorption to 1.46 and 2.40 mg/g under mixotrophic and microoxic conditions, respectively. Removal efficiency from the solution was below 9% at pH 2.5 and up to 86% at pH 5.0 (Fig. S1).

Individual metal sorption data revealed no particular selectivity towards REEs over Cu under any condition (Fig. 1c). In fact, sorption of Cu by mol was 1.7–10 × greater than any REE, though this might in part be explained by the higher molar starting concentration of Cu compared to the REEs.

Microoxic conditions resulted in overall higher biomass-specific metal sorption compared to mixotrophic conditions (Fig. 1a, c). However, biomass concentration under microoxic conditions decreased by 21–25% after 20 h, whereas growth by 18–28% could be observed in mixotrophic cultures (Fig. 1b). This indicates that increased biosorption is not necessarily a result of higher metabolic activity or cell health.

### Biosorption of REEs in spiked growth media

To assess REE biosorption under conditions supporting metabolic activity and growth of acidophiles, we spiked cultures of *G. sulphuraria* in nutrient replete medium with La. Experimental conditions at the beginning and end of the tests are shown in Table 1. Initial experiments conducted at initial pH 1.5 did not result in any La biosorption under any growth conditions. We therefore tested whether biosorption occurs at higher pH (thus, reduced cell surface protonation) and at a higher La equilibrium concentration. No biosorption was observed under these conditions either, despite apparent metabolic activity and growth.

**Table 1:** Initial and final La (*c*_La,0_,* c*_La,end_) and biomass (*c*_X,0,_ *c*_X,end_) concentrations from biosorption experiments with *G. sulphuraria* in *Galdieria* growth media spiked with La_2_(SO_4_)_3_

pH_0_^a^	Growth conditions	Duration (days)	*c*_La,0_ (µM)	*c*_La,end_ (µM)	*c*_X,0_ (g/L)	*c*_X,end_ (g/L)
1.5	Heterotrophic	6	21.0 ± 0.6	21.6 ± 1.1*	0.20 ± 0.01	3.00 ± 0.15
1.5	Mixotrophic	6	19.8 ± 0.5	19.9 ± 0.6*	0.20 ± 0.01	4.57 ± 0.21
1.5	Phototrophic	18	13.3 ± 0.1	13.4 ± 0.1*	0.21 ± 0.00	1.24 ± 0.07
2.5	Phototrophic	14	96.3 ± 2.5	100 ± 1*	1.07 ± 0.04	1.56 ± 0.04

^a^Initial pH; pH at the end of the experiments was 1.4 and 2.2 for pH_0_ 1.5 and 2.5, respectively

*No significant reduction from initial La concentration, *c*_La,0_ (Welch’s one-sided *t*-test, *α* = 0.05)

### REE solubility in the presence of phosphate

We subsequently assessed the REE solubility and feasibility of REE biosorption at less acidic but nutrient-replete conditions (i.e., in the presence of phosphate) by means of thermodynamic modelling using OLI StreamAnalyser v.11.5.

**Fig. 2:**
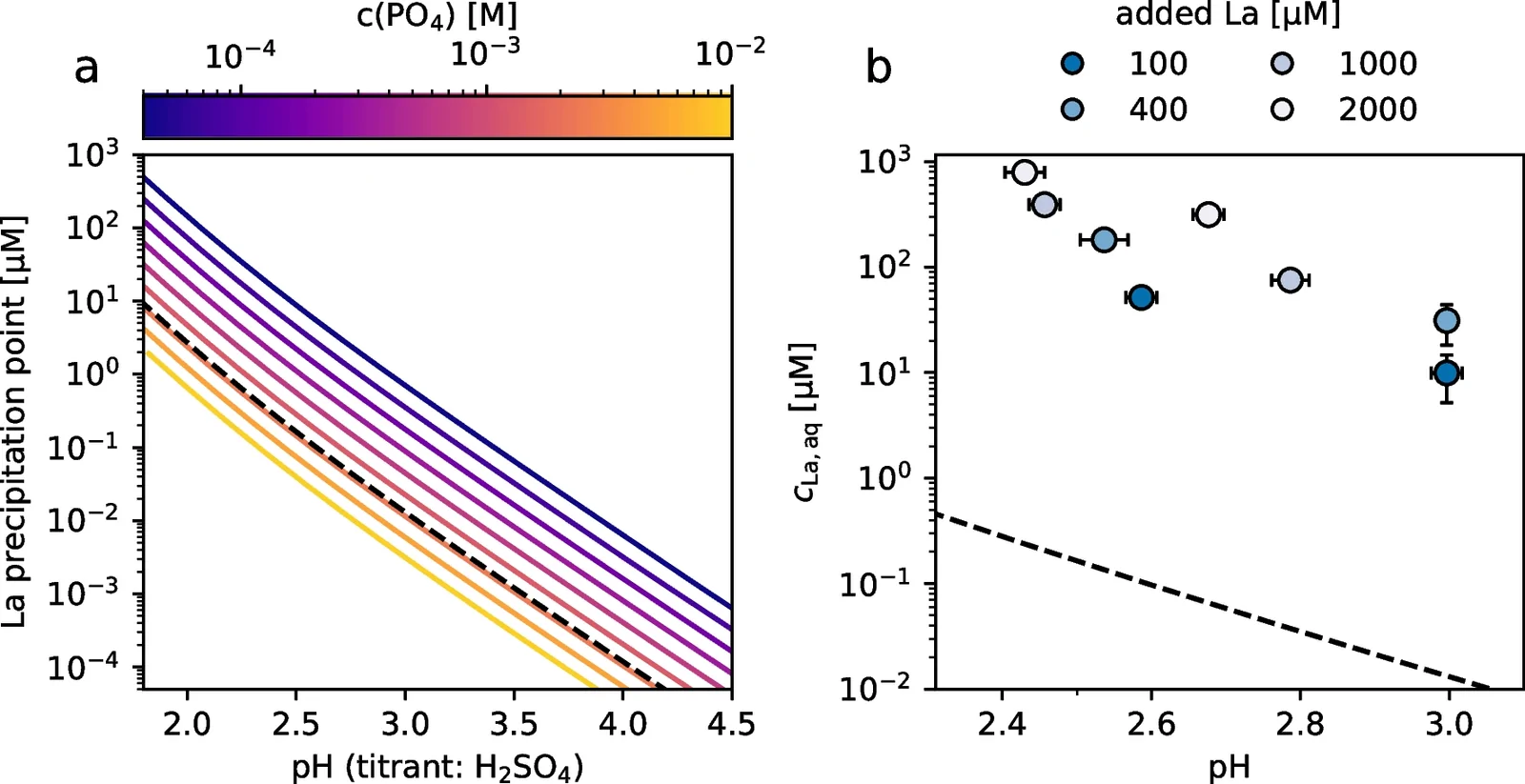
Predicted solubility of LaPO_4_ • x H_2_O (**a**) and measured dissolved La (**b**) in *Galdieria* medium. The dashed lines represent simulation results at a phosphate concentration of 2.2 mM, which is that of *Galdieria* medium used in experiments. Data shown in **b** is from experiments with added seeds, 72 h after addition of LaCl_3_

Figure 2a shows the simulated precipitation points of LaPO_4_ • x H_2_O at varying pH and phosphate concentrations in the growth media used for cultivation of *G. sulphuraria*. The theoretical solubility of La is low as expected. At a phosphate concentration of 2.2 mM (that of the used *Galdieria* medium) and pH = 2.5, the predicted precipitation point by OLI is at 0.2 µM La and drastically decreases with increasing pH.

**Fig. 3:**
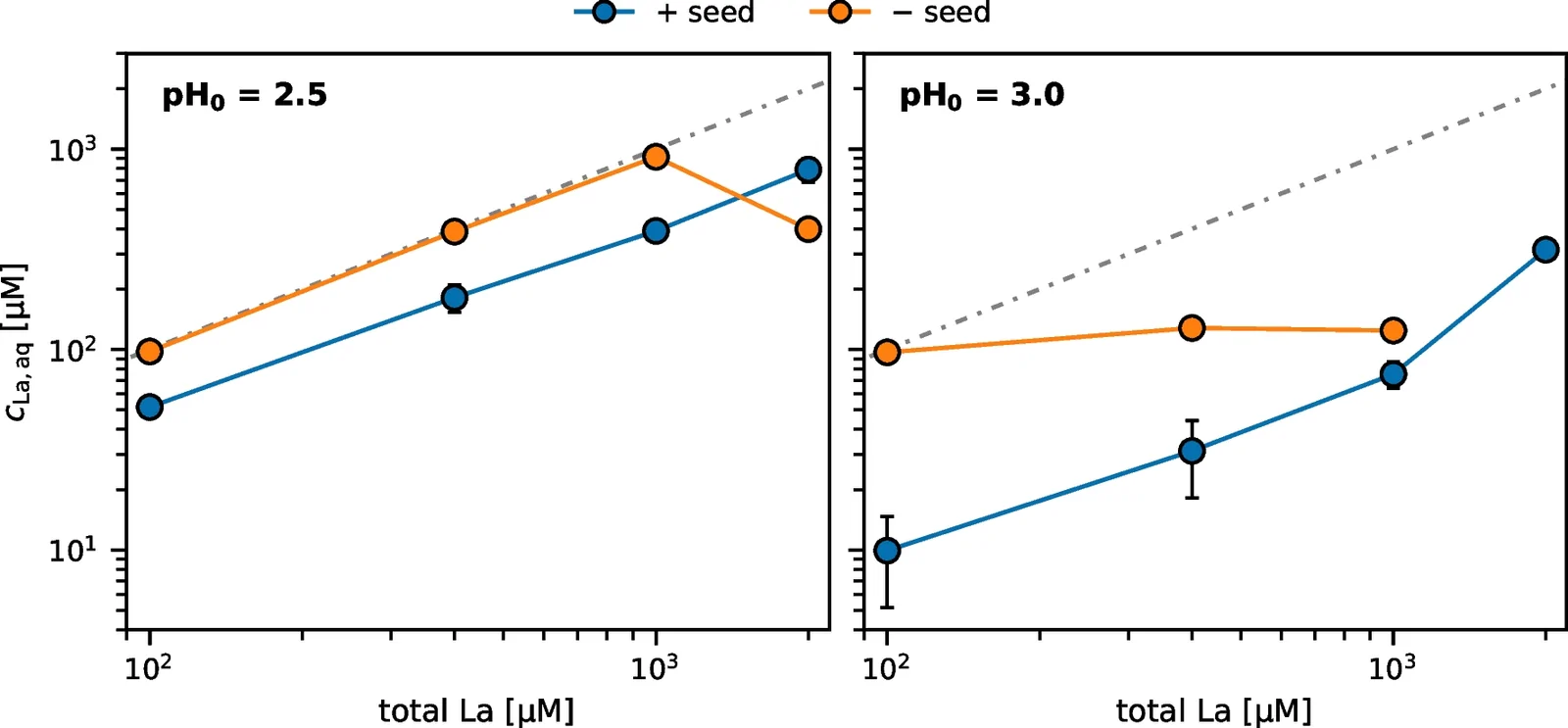
Impact of addition of seeds on dissolved La concentrations in *Galdieria* medium 72 h after adding LaCl_3_ at two initial pH values. The dash-dotted reference line represents theoretical 100% dissolved La

The experimentally determined concentrations of dissolved La in *Galdieria* medium were substantially higher than predicted. In solutions with added seeds, dissolved La ranged from 9.9 to 791 µM at a final pH of 3.0 and 2.5, respectively (Fig. 2b). This indicates that the solutions were supersaturated and not at thermodynamic equilibrium after 72 h. This was particularly evident in solutions without added seeds, where the addition of up to 1000 µM La at initial pH 2.5 (final pH 2.4) and up to 100 µM La at initial pH 3.0 (final pH 3.0) did not result in any precipitation, whereas the addition of seeds led to a reduction of dissolved La concentrations by up to 10x (Fig. 3). Furthermore, the weakly crystalline characteristics of the precipitated phase shown by X-ray diffraction analysis (Fig. S2) points to its metastable state. Lastly, dissolved La concentrations were reduced by only 15–33% after 7 more days (Fig. S3), indicating slow kinetics of LaPO_4_ precipitation under the studied conditions.

La solubility was also assessed in other growth media that have previously been used in studies for REE biosorption with microalgae (Fischer et al. 2019; Fritz et al. 2022a, b; Kim et al. 2022) (Table 2). In Bold’s basal medium at pH 6.8, precipitation reduced aqueous La concentration below detection, which was expected at circumneutral conditions. The behavior of La in the acidic Hutner and yeast extract media was similar to that observed in the *Galdieria* medium: La remained completely dissolved when no seed was added but dropped by 33–41% in the presence of seed solids. Moreover, La readily and completely precipitated in the KH_2_PO_4_ controls, indicating a masking effect of other medium components that reduces precipitation of LaPO_4_.

**Table 2:** La solubility in various growth media previously used in biosorption studies. Solutions were spiked with 100 µM LaCl_3_ and equilibrated for 96 h at room temperature before measuring for dissolved La

Matrix	*c*_PO4,0_ (mM)	pH_0_	*c*_La,end_ (µM)	Reference
H_2_O control	0	5.5	97.3 ± 1.2	−
Bold’s basal medium	1.7	6.8	< L.O.D.^a^	Fischer et al. (2019), Fritz et al. (2022a,b)
Hutner medium, − seed	2.9	3.3	96.1 ± 0.4*	Kim et al. (2022)
Hutner medium, + seed	2.9	3.3	49.7 ± 9.3**	
KH_2_PO_4_ control	2.9	3.3	< L.O.D.^a^	
Yeast extract, − seed	> 0.24^b^	3.3	86.0 ± 8.3*	
Yeast extract, + seed	> 0.24^b^	3.3	64.7 ± 7.0**	
KH_2_PO_4_ control	0.24	3.3	< L.O.D.^a^	

^a^Detection limit

^b^Actual *c*_PO4_ was not determined but equals the sum of phosphate from yeast extract and KH_2_PO_4_

*No significant difference compared to control (Welch’s one-sided *t*-test, *α* = 0.05)

**Significant difference compared to control (Welch’s one-sided *t*-test, *α* = 0.05)

## Discussion

### REE biosorption with *G. sulphuraria*

There has been considerable attention towards the use of acidophilic microalgae for biosorption of REEs or other transition metals, specifically *Euglena* (Khatiwada et al. 2020; Jasso-Chávez et al. 2021; Kim et al. 2022; Zak et al. 2023) and *Galdieria* (Minoda et al. 2015, 2022; Iovinella et al. 2022; Singh et al. 2023). The acidophilic red alga *G. sulphuraria* has particularly gained attention for REE biosorption after Minoda et al. (2015) showed efficient removal (> 90%) of low concentrations of La, Nd, and Dy (5 ppm each) from metal solutions without nutrient salts. Based on their reported data on removal efficiency, we estimated the corresponding total REE sorption capacity to be approximately 1.3 mg/g at pH 1.5–2.5 under microoxic conditions, compared to 0.29 mg/g in the present study. Notably, they reported approximately twofold higher REE sorption at pH 1.5–2.5 than at pH 5.0. This stands in contrast to our results as well as the majority of literature on biosorption, specifically with *G. sulphuraria* (Iovinella et al. 2022; Manfredi et al. 2023), as well as other biosorbents, where sorption is commonly reduced under highly acidic conditions (Gupta et al. 2019; Mattocks and Cotruvo 2020). This is due to the protonation of binding sites causing reduced charge-based and acid–base interactions. A possible explanation for the reduced REE sorption at pH 5.0 in the study of Minoda et al. (2015) is the use of high concentrations of MES (0.2 M) as a buffer. It has been shown that MES scavenges significant amounts of Eu^3+^ via complexation at buffer concentrations of 10 mM (Mandal et al. 2022). Thus, lanthanide ions are likely unavailable for adsorption at MES concentrations as high as 0.2 M.

We showed that REE biosorption with *G. sulphuraria* is not efficient under conditions that simultaneously allow sustained growth, namely, high acidity (pH 1.5–2.5) and the presence of phosphate. Under nutrient-limiting conditions, decaying cells even sorbed more metal than growing ones. Indeed, metabolic activity can reduce biosorption by acidophiles, as they have to selectively exclude, sequester, and detoxify abundant harmful metals in their natural environment. *G. sulphuraria* and other acidophiles possess membrane transporters that aid in keeping cytosolic levels of non-essential metals (including lanthanides) low, in part by efflux into the extracellular space (Schönknecht et al. 2013; Johnson and Aguilera 2016; Khatiwada et al. 2020). Furthermore, acidophiles maintain a positive membrane potential by actively pumping K^+^ into the cytosol, which may further reduce the influx of cations into the cell (Johnson and Aguilera 2016). Thus, a reduction in ATP levels under microoxic conditions (Lafraie and Betz 1985) and concomitant reduction in efflux activity and membrane potential might have caused increased REE sorption. The high metal tolerance of extreme acidophiles therefore further challenges the feasibility of simultaneous biosorption and self-replication under ideal growth conditions. Nonetheless, their defense mechanisms can still be harnessed: acidophilic organisms, particularly when growing in biofilms, produce high amounts of extracellular polymeric substances (EPS) rich in acidic functional groups (Aguilera et al. 2008; Naveed et al. 2019). Zak et al. (2023) showed that EPS significantly contribute to REE sorption capacity at pH 5.0. The biofilm-derived biomass used in their study reached La sorption capacities of over 60 mg/g, which is over 20 times higher than REE sorption with *G. sulphuria* in this study.

Efficient REE biosorption at low pH (≈2.5–3.0) has recently been demonstrated with immobilized lanmodulin (Dong et al. 2021). This small protein is involved in lanthanide trafficking in methylotrophic bacteria and has picomolar affinity towards REEs (Deblonde et al. 2020), enabling selective purification from complex mixtures (Dong et al. 2021). However, the high cost of protein purification is a challenge for scale-up. Other recently proposed strategies to utilize the remarkable affinity of lanmodulin for REEs include surface display of the protein on microorganisms (Xie et al. 2022), or using methylotrophic bacteria with lanthanide-dependent metabolic pathways for bioaccumulation directly in the waste stream (Singer et al. 2023).

### The role of lanthanide phosphates in biosorption processes

We confirmed that orthophosphate limits the solubility of REEs in various media and, thus, their availability for biosorption, particularly at low acidity. On the contrary, Fischer et al. (2019) reported virtually complete REE biosorption with cyanobacteria *Anabaena* from nutrient-replete Bold’s basal medium at pH ≈ 7. They claimed that no precipitation occurred (contrary to our results and thermodynamic models) without providing a quantitative control. However, dissolution of REEs in the algal culture might have occurred due to secreted EPS from *Anabaena* (Freire-Nordi et al. 2005).

REE phosphate precipitation also constrains simultaneous biosorption and growth under acidic conditions, as we showed at pH as low as 2.5, though aqueous REE concentrations were up to three orders of magnitude higher than expected. The large disparity between predicted La solubility and measured dissolved La concentration is explained by supersaturation, which might have been caused by high concentrations of complexing agents, such as sulfate (Kim and Osseo-Asare 2012) in *Galdieria* medium, malate (Rasoulnia et al. 2021) in Hutner medium, or the complex mixture of proteins and carbohydrates in yeast extract. This phenomenon needs to be considered in biosorption studies to avoid spurious claims about the role of biomass in REE recovery. For example, Horiike et al. (2016) showed that the fungus *Penidiella* sp. recovered Dy from an acidic growth medium (pH = 2.5) containing 0.65 mM phosphate and 100 ppm Dy as DyPO_4_ precipitates. This system might have been supersaturated with Dy, so that precipitation of Dy with orthophosphate from the surrounding medium was induced by cells acting as nucleation sites, rather than metal binding sites. Similarly, Kim et al. (2022) used Hutner media and stream water enriched with yeast extract to study REE biosorption in *Euglena gracilis*. While we showed that La precipitated in these media after the addition of nucleation LaPO_4_ seeds, the above authors did not include similar control experiments, making conclusions about the mechanism of REE removal uncertain. It thus becomes clear that biosorption and bioaccumulation studies require proper consideration of the experimental matrix, in order to enable solid conclusions about the true fate of REEs.

In summary, we showed that the acidophilic alga *G. sulphuraria* biosorbs REEs significantly better at mildly acidic conditions (pH 5.0, *q*_REE_ up to 2.40 mg/g) than at the extremely acidic conditions (pH ≤ 2.5, *q*_REE_ up to 0.29 mg/g) it is adapted to in its natural environment. Extrapolating these findings generally to acidophilic microalgae, which possess no known REE-specific ligands, we conclude that their innate metal tolerance hinders REE sorption under ideal acidic growth conditions (pH < 3), although the biosorption properties of their exterior cell wall and EPS are functioning better as ligands under less acidic conditions because they are likely deprotonated. We further conclude that for biosorption to occur, phosphate-limiting conditions should prevail, even at pH as low as 2.5. In dilute REE-containing solutions, algal growth should occur separately from a biosorption process step, due to the low solubility constants of REE phosphates. Finally, we stress the importance of conducting REE biosorption or bioaccumulation studies under properly controlled experiments that separate REE biosorption from REE precipitation and consider solution matrix interactions as well potential supersaturation effects to ensure reliable determination of REE biosorption.

## Supplementary Information

Below is the link to the electronic supplementary material.

Supplementary file1 (PDF 246 KB)

## Data Availability

The data that support the findings of this study are available from the corresponding author upon reasonable request.
