# Supplementary material for: Roles of pH and phosphate in rare earth element biosorption with living acidophilic microalgae
Source: Appl Microbiol Biotechnol. 2024 Mar 14;108:262. doi: 10.1007/s00253-024-13068-8 (PMC10940408; doi:10.1007/s00253-024-13068-8)
Supplement: Supplementary file 1 [file 253_2024_13068_MOESM1_ESM.pdf]

Supplementary Information to:

Roles of pH and Phosphate in Rare Earth Element Biosorption with Living Acidophilic Microalgae

Jens Kastenhofer[1], Oliver Spadiut[2], Vladimiros G. Papangelakis[1], D. Grant Allen[1*]

[1] University of Toronto, Department of Chemical Engineering and Applied Chemistry, 200 College Street, Toronto, ON M5S 3E5, Canada

[2] TU Wien, Institute of Chemical, Environmental and Bioscience Engineering, Research Division Biochemical Engineering, Research Group Integrated Bioprocess Development, Gumpendorfer Straße 1a, 1060 Vienna, Austria

* Corresponding author: D. Grant Allen, dgrant.allen@utoronto.ca

Supplementary Text 1

To synthesize solid $LaPO_4$, solutions of 1 M $LaCl_3$ and 1 M NaH_2PO_4 were prepared and 2.5 mL each were transferred to a 15 mL polypropylene tube. White precipitate formed immediately and the generated suspension was mixed on a vortexer for 5 minutes. The precipitated $LaPO_4$ was then aged for 24 hours at 25°C. Subsequently, the suspension was centrifuged for 5 min at 10000 × g and 21°C and the solids were washed twice with ultrapure water. Solid $LaPO_4$ was then resuspended in ultrapure water, transferred to a glass petri dish, and dried in an oven at 95°C.

Table S1 Composition of modified Bold's Basal Medium

	Stock concentration [g/L]	Added per 1 L medium [mL]		Stock concentration [g/L]	Added per 1 L medium [mL]
$NaNO_3$	250	1	Trace compound stock containing:		1
KH_2PO_4	175	1	H_3BO_3	2.86	
K_2HPO_4	75	1	$ZnSO_4 \cdot 7\ H_2O$	0.22	
$MgSO_4 \cdot 7\ H_2O$	75	1	$Na_2MoO_4 \cdot 2\ H_2O$	0.39	
CaCl2 • 2 H2O	25	1	$CuSO_4 \cdot 5\ H_2O$	0.08	
NaCl	25	1	$Co(NO_3)_2 \cdot 6\ H_2O$	0.05	
$Na_2EDTA \cdot 2\ H_2O$ (+ KOH)	10 (6.2)	1			
$FeSO_4 \cdot 7\ H_2O$	4.98	1			
H_3BO_3	11.5	0.7			

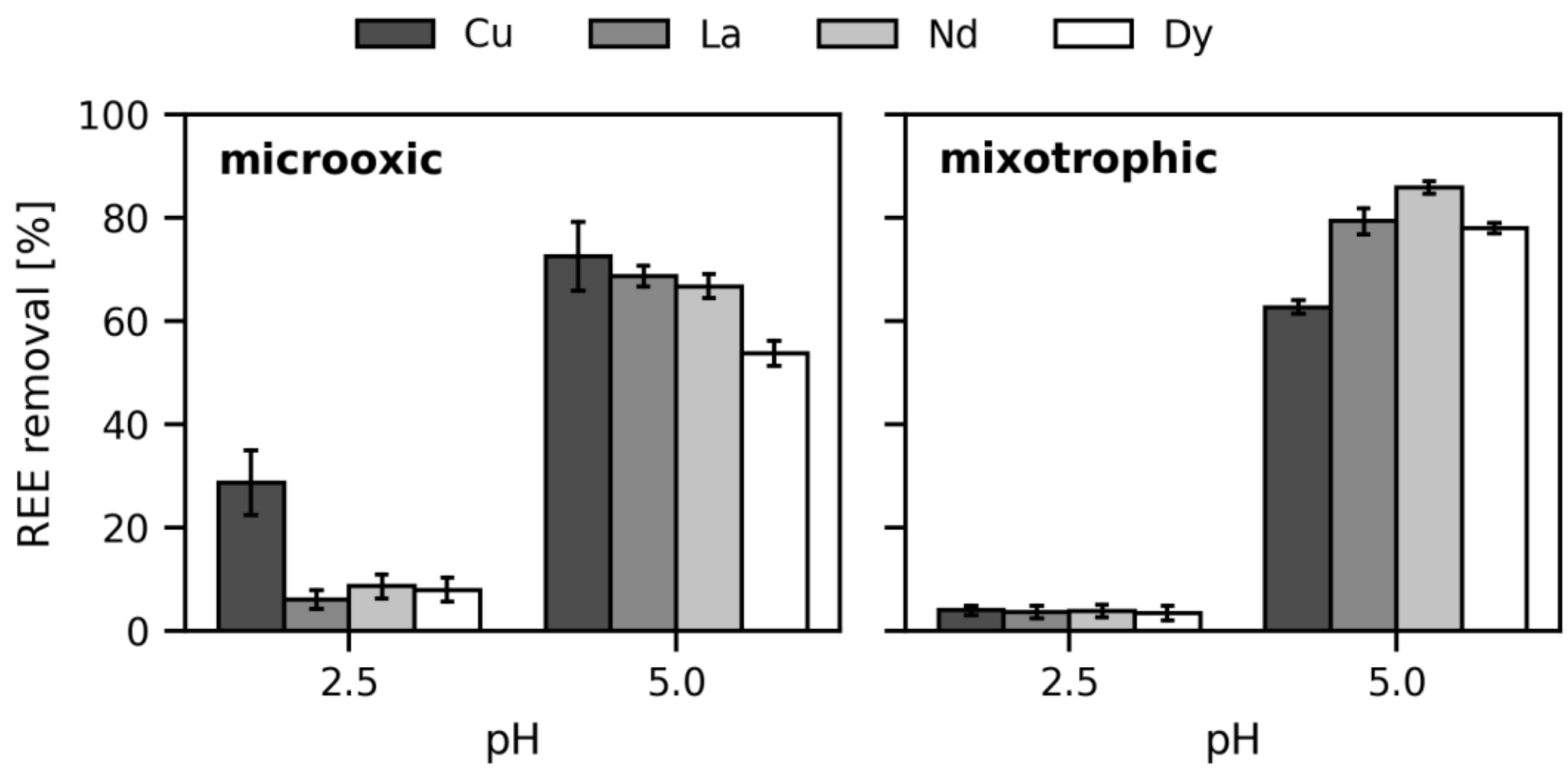

Fig. S1 Efficiency of REE removal with *G. sulphuraria* from an acidic sulfate system containing 5 ppm each of Cu (79 µM), La (36 µM), Nd (35 µM), and Dy (31 µM).

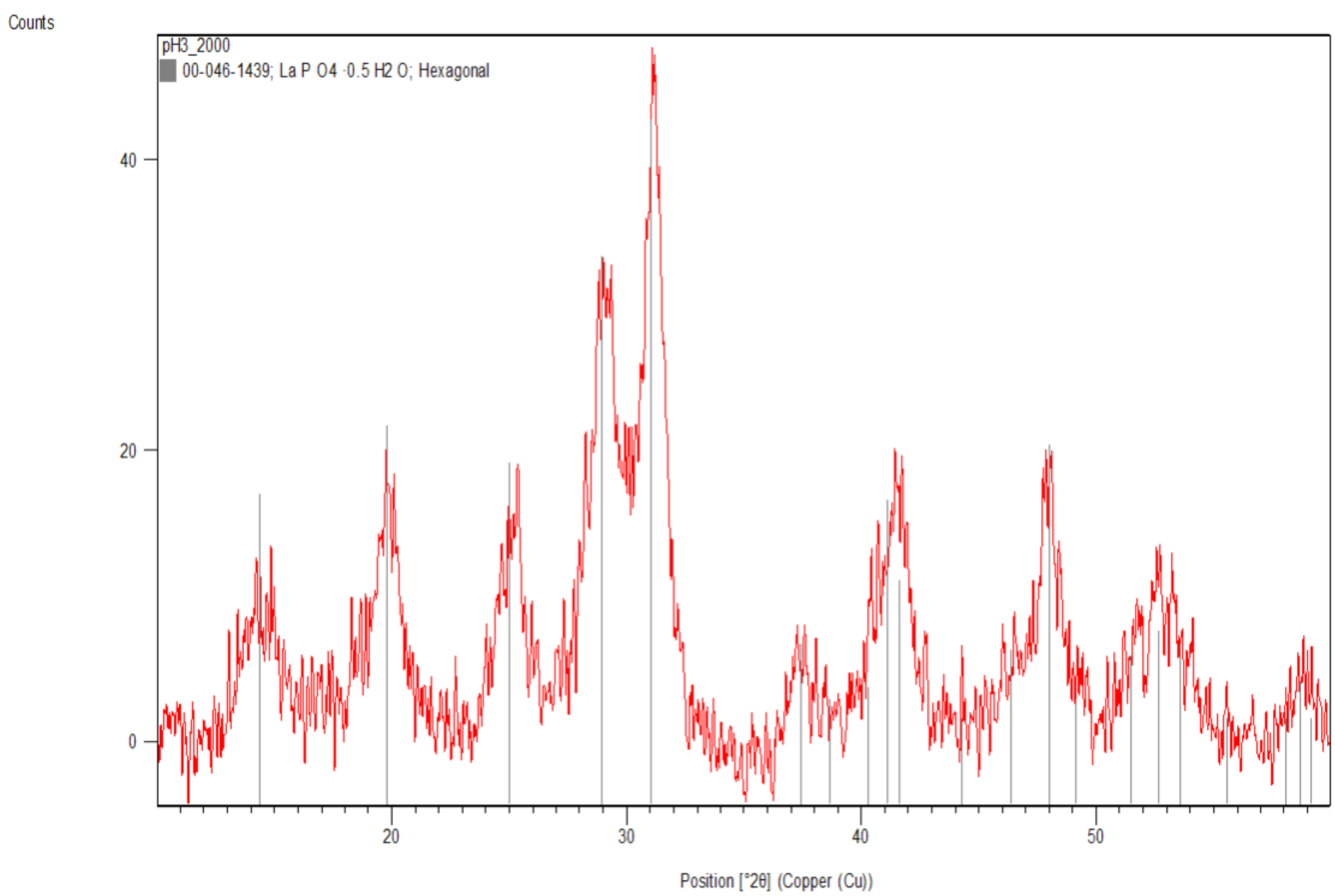

Fig. S2 XRD pattern of the precipitate formed in *Galdieria* medium at initial pH = 3, 72 h after spiking the solution with 2000 µM $LaCl_3$. The grey lines are the reference pattern for crystalline $LaPO_4 \cdot 0.5\ H_2O$. The XRD pattern of the sample displays poorly defined peaks, indicating weak crystallinity.

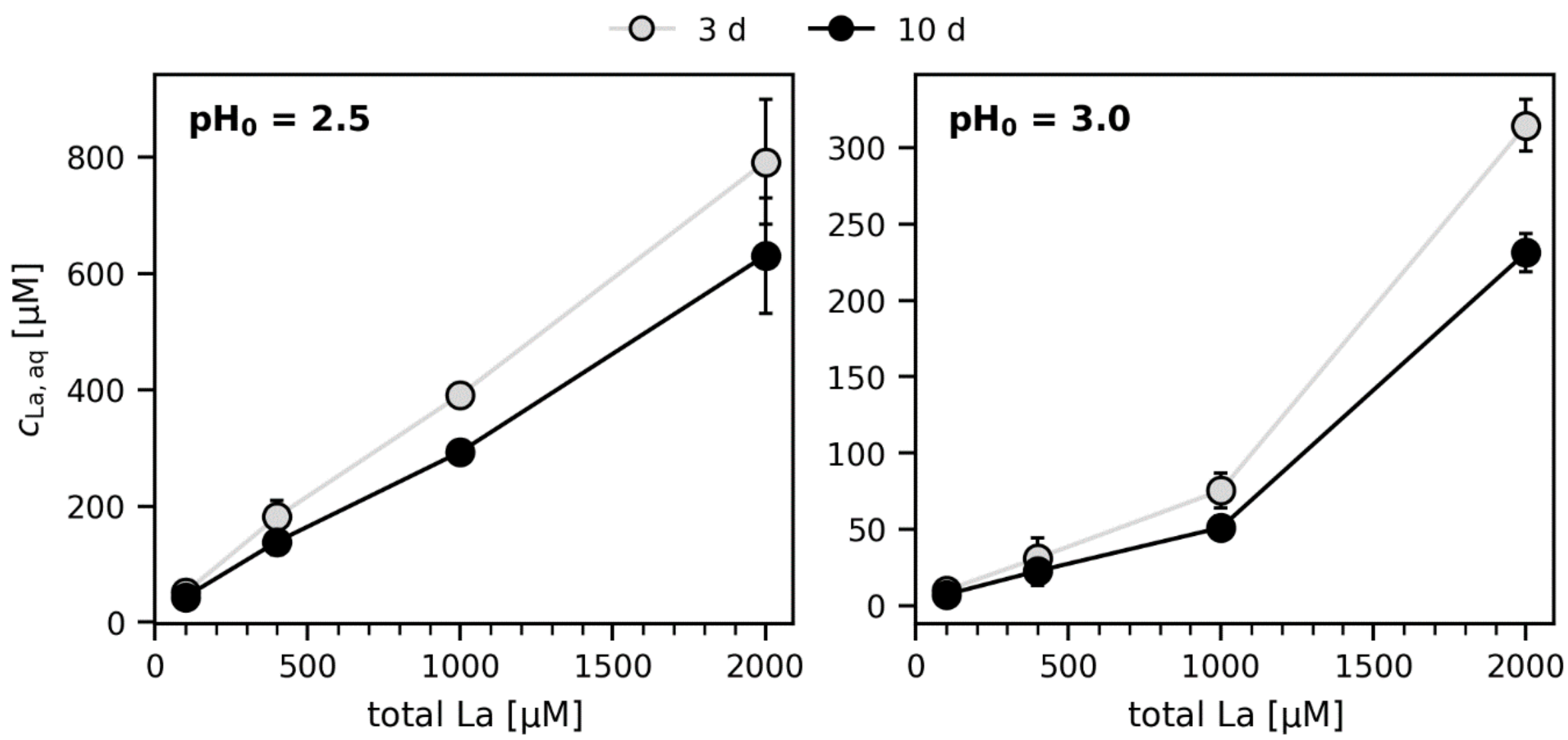

Fig. S3 Dissolved La concentrations in *Galdieria* medium at two initial pH values, 3 and 10 days after adding $LaCl_3$.
